# Longitudinal profiling of the blood transcriptome in an African green monkey aging model

**DOI:** 10.18632/aging.202190

**Published:** 2020-12-03

**Authors:** Ja-Rang Lee, Se-Hee Choe, Young-Hyun Kim, Hyeon-Mu Cho, Hye-Ri Park, Hee-Eun Lee, Yeung Bae Jin, Ji-Su Kim, Kang Jin Jeong, Sang-Je Park, Jae-Won Huh

**Affiliations:** 1 Primate Resource Center, Korea Research Institute of Bioscience and Biotechnology, Jeongeup 56216, Republic of Korea; 2 National Primate Research Center, Korea Research Institute of Bioscience and Biotechnology, Cheongju 28116, Republic of Korea; 3 Department of Functional Genomics, KRIBB School of Bioscience, Korea University of Science and Technology (UST), Daejeon 34113, Republic of Korea

**Keywords:** African green monkey, aging, aging candidate gene, longitudinal transcriptome

## Abstract

African green monkeys (AGMs, *Chlorocebus aethiops*) are Old World monkeys which are used as experimental models in biomedical research. Recent technological advances in next generation sequencing are useful for unraveling the genetic mechanisms underlying senescence, aging, and age-related disease. To elucidate the normal aging mechanisms in older age, the blood transcriptomes of nine healthy, aged AGMs (15‒23 years old), were analyzed over two years. We identified 910‒1399 accumulated differentially expressed genes (DEGs) in each individual, which increased with age. Aging-related DEGs were sorted across the three time points. A major proportion of the aging-related DEGs belonged to gene ontology (GO) categories involved in translation and rRNA metabolic processes. Next, we sorted common aging-related DEGs across three time points over two years. Common aging-related DEGs belonged to GO categories involved in translation, cellular component biogenesis, rRNA metabolic processes, cellular component organization, biogenesis, and RNA metabolic processes. Furthermore, we identified 29 candidate aging genes that were upregulated across the time series analysis. These candidate aging genes were linked to protein synthesis. This study describes a changing gene expression pattern in AGMs during aging using longitudinal transcriptome sequencing. The candidate aging genes identified here may be potential targets for the treatment of aging.

## INTRODUCTION

Aging is defined as progressive multifactorial functional decline over time at the molecular, cellular, tissue, and organismal levels [1]. The aging organism becomes frail, with increased disease susceptibility. Aging is a major risk factor for aging-related diseases including neurodegeneration, cardiovascular disease, osteoporosis, and cancer [2]. This process depends on the interaction between numerous genetic, environmental, and lifestyle factors [3]. The individual genetic or epigenetic background affects cellular senescence. Consequently, the genetic and epigenetic backgrounds are the main factors related to aging within organisms. Molecular mechanisms of aging can be attributed to accumulated genetic mutations and epigenetic dysfunction [4]. These molecular alterations interact directly with the transcriptional network. Thus, identifying aging-related molecular features has critical implications for enhancing our understanding of aging and the mechanism underlying aging-induced disease.

Because peripheral whole blood sampling is easy to access and non-invasive, researchers use whole blood to investigate molecular profiles and disease-associated molecular biomarkers [5]. This approach is based on the idea that gene expression in peripheral whole blood reflects expression profiles related to pathological changes in other tissues [6]. To fully explore the information contained in the molecular blood signature, molecular profiling tools using -omics analyses were applied to whole blood, giving rise to the field of “bloodomics” [7]. Numerous investigators apply bloodomics assays in studies on aging [8–11]. Large-scale analyses of the relationship between aging and gene expression have been performed using human peripheral blood samples [9, 12]. Most studies are cross-sectional; however, several longitudinal studies focused on specific aging-related hypofunction [13], only used short-term observations [14], or examined specific cell populations [15, 16]. While these massive scale studies provide insights into the complex biological processes associated with human aging, transcriptome studies involving natural aging over time using identical samples and controlled environmental conditions remain lacking.

To date, studies on aging have laid extensive groundwork using models such as worms, flies, fish, and rodents. These models provide insights into the process of aging. However, for clinical applications, more complex animal models such as non-human primates (NHPs), which exhibit aging mechanisms that are similar to those observed during human aging, are required [17]. NHPs share genetic, physiological, immunological, and behavioral similarities with humans. In addition, inter-individual variation in NHPs is similar to the variation observed in humans, and NHP models recapitulate naturally-occurring age-associated diseases even in the absence of genetic manipulation [18, 19]. NHP studies permit complete control of environmental exposures including diet, environmental factors, housing, and social interactions. Therefore, longitudinal NHP studies can overcome the limitations associated with other models of aging and human studies. Thus, longitudinal NHP studies provide an excellent opportunity to study the actual mechanisms involved in aging over time.

The African green monkey (AGM) is a medium-sized Old World monkey (OWM) that originated in Africa. AGMs have an average lifespan of 11-13 years in the wild, while they can survive for more than 31 years in captivity [19]. Traditionally, AGMs are used as an experimental NHP model for biomedical research [20], particularly as models of infectious disease, human immunodeficiency virus, and preclinical pharmacokinetic studies [21]. They are also important models of neurological, degenerative, and cardiovascular disease, and cognitive and social behavior. AGMs exhibit the same or similar degenerative diseases as humans, including cancer, dementia, Parkinson’s disease, and cardiovascular impairments. For these reasons, AGMs serve as an excellent animal model for studying changes related to aging and age-associated disease. In addition, the draft genome of AGM was recently published and is now available in GenBank (AGM GenBank Assembly ID, GCA_000409795.2). Therefore, the above-mentioned characteristics make AGMs an attractive animal model in aging-related studies.

Here, we performed a longitudinal gene expression study in healthy aged AGMs (n=9) by obtaining peripheral blood cells at three time points over a period of two years using non-invasive biopsy. To identify the most relevant gene expression signatures for aging over time, we performed transcriptomic analysis at three time points and compared differentially expressed genes (DEGs) between two time points. Furthermore, candidate aging genes were selected by time series analysis. Gene ontology (GO) and gene interaction analyses showed that upregulated genes or candidate aging genes are associated various biological processes, with ribosome and translation processes among the most represented.

## RESULTS

### Global transcriptional profiling and clustering

To compare the temporal transcriptional changes, samples from nine naturally-aged AGMs (age 15 to 23, and consisting of seven females and two males) were analyzed by RNA-seq over a period of two years. Samples from each animal were collected at three consecutive time points (Supplementary Figure 1 and Supplementary Table 1). About 43.1-62.3 million raw reads from each sample were sequenced (Supplementary Table 2). After the low-quality reads were filtered out, 82.3-90.0% reads were analyzed and uniquely mapped to the AGM ChlSab1.1 reference genome. The expression of each gene was normalized to fragments per kb per million fragments (FPKM); the FPKM values were considered the final level of expression for each gene. We calculated Pearson’s correlation coefficients to compare global gene expression between samples (Supplementary Table 3). The intra-individual correlation coefficients decreased over time. Furthermore, the number of high inter-individual correlation coefficients exhibited a time-dependent decrease at the second and third time points compared with the first time point.

**Figure 1:**
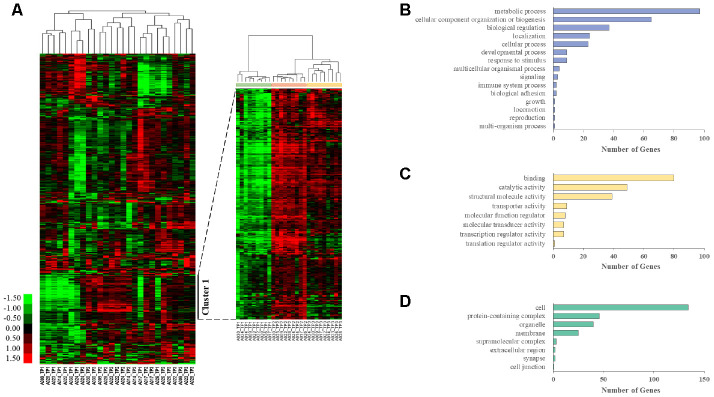
**Longitudinal aging-associated global expression profiling.** (**A**) Hierarchical clustering is presented in a matrix format, where each row represents an individual gene and each column represents a different time point in each AGM. Red, high expression; green, low expression. Cluster 1 contains the genes that are highly expressed in TP2 and TP3 compared with those in TP1. PANTHER GO slim enrichment analysis of (**B**) biological processes (BP), (**C**) molecular function (MF), and (**D**) cellular components (CC).

We also performed unsupervised clustering analysis and generated heat maps from whole transcriptome data using Cluster 3.0 software. Genes with an FPKM value above 2^5^ in at least one sample and a fold change greater than 2 between the highest and lowest samples were excluded to remove noninformative genes for clustering. This resulted in 1866 unique genes. Hierarchical clustering analysis identified gene groups at each time point (Figure 1A). Cluster 1 contained 289 genes, and the correlation between genes in the cluster was 0.52. Then, Cluster 1 was annotated using the PANTHER (Protein Analysis through Evolutionary Relationships) classification system, which identified 272 mRNAs distributed in various GO categories. Among the GO categories, metabolic processes, cellular component organization or biogenesis, biological regulation, binding, and cell terms were dominant (Figure 1B–1D).

### Characterization of individual aging processes in aged AGMs

**Figure 2:**
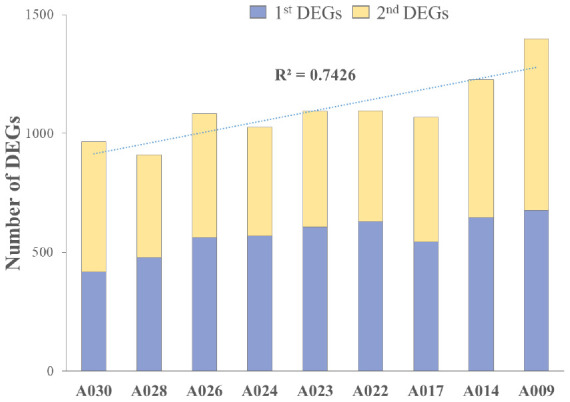
**Cumulative individual DEGs in aged AGMs.** The number of DEGs increased with chronological age. A030 to A009 were in reverse order of chronological age. A030 was the youngest, while A009 was the oldest.

**Figure 3:**
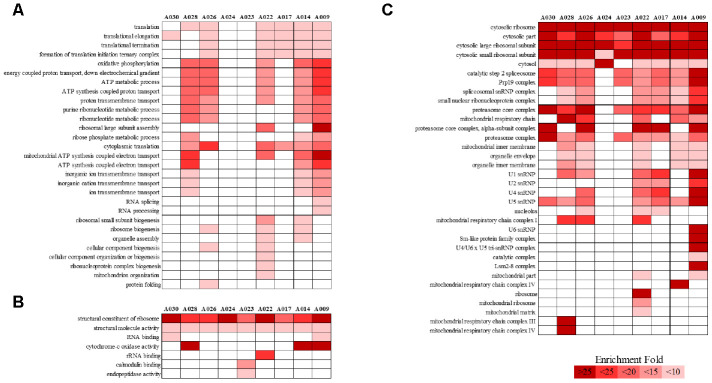
**PANTHER GO slim analysis of individual aging-related DEGs.** (**A**) BP, (**B**) MF, and (**C**) CC enrichment. Each GO term was sorted using the following parameters: –log_10_(p-value) > 2.5 and enrichment > 5. Gradient colors indicate the degree of enrichment.

We analyzed RNA-seq data from whole-blood samples of aged AGMs. Then, we selected genes that were differentially expressed over time for each individual. Genes that satisfied the following two conditions were considered as DEGs: (1) *p*-value < 0.05, and (2) |fold change| ≥ 1.3. The DEGs were compared as follows: (1) time point (TP) 2 vs TP 1; (2) TP 3 vs TP 2; and (3) TP 3 vs TP 1. To investigate aging in each individual, we compared the number of DEGs in each aged AGM. Linear regression analysis of DEGs showed that the number of DEGs tended to increase with age (Figure 2). Then, we identified the aging-related DEGs using Venn diagram analysis (Supplementary Figure 2). The aging-related DEGs were defined as overlapping genes in each DEG set. Then, the aging-related DEGs were annotated using the PANTHER classification system for GO analysis. In this analysis, GO terms with a corrected *p*-value < 0.05 were considered significantly enriched. Then, we selected GO terms with two criteria: i.e., -log(*p*-value) > 2.5 and enrichment fold > 5. When we analyzed the upregulated aging-related DEGs, we found significant enrichment in translation- and ATP synthesis-related biological processes in more than half of all the aged AGMs (Figure 3A). The molecular functions, “structural ribosome constituent” and “structural molecule activity” were overrepresented in all aged AGMs (Figure 3B). The most overrepresented cellular components were “ribosome,” “ribosomal subunit,” “cytosol,” “spliceosome,” “proteasome,” and “mitochondrial membrane” in all or more than half of the aged AGMs (Figure 3C). Individual GO terms were “RNA splicing” and “protein folding associated with protein quality control” (Figure 3A). In the case of downregulated genes, significant GO-term enrichment was not detected.

### Characterization of common aging-related genes in aged AGMs

**Figure 4:**
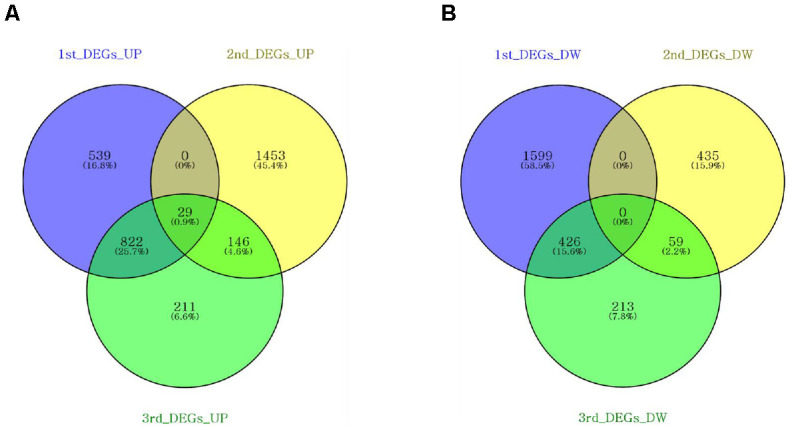
**Venn diagrams showing the DEG overlap from pairwise comparisons of each time point.** Venn diagrams were constructed using Venny online software. (**A**) Nine hundred and ninety-seven upregulated genes were shared across DEG sets. (**B**) Four hundred and eighty-five downregulated genes were shared across the DEG sets.

**Figure 5:**
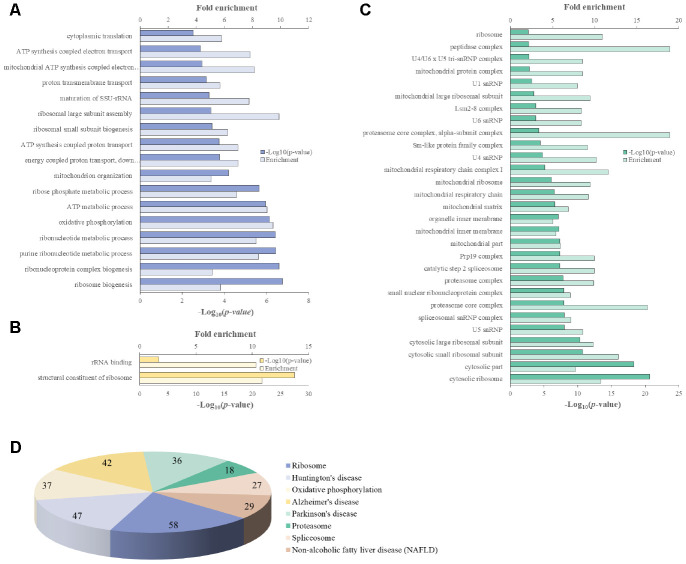
**PANTHER GO slim analysis and KEGG pathway analysis of common aging-related DEGs.** (**A**) BP, (**B**) MF, (**C**) CC enrichment, and (**D**) KEGG pathways. Each GO term and KEGG pathway were sorted using the following parameters: –log_10_(p-value) > 2.5 and enrichment > 5.

We attempted to identify the common aging-related gene expression patterns among nine aged AGMs. Common DEGs over time were selected using the same criteria used to select individual DEGs. Each individual was regarded as a biological replicate. To identify the common aging-related DEGs, we performed Venn diagram analysis (Figure 4). The common aging-related DEGs were defined as overlapping genes in each DEG set. We identified 997 upregulated genes, which were annotated using the PANTHER classification system (Figure 4A). We selected GO terms using two criteria, i.e., -log(*p*-value) > 2.5 and enrichment fold > 5. The highest overrepresented biological processes were “ribosome biogenesis,” “ribonucleoprotein complex biogenesis,” “purine ribonucleotide metabolic process,” “oxidative phosphorylation,” and “ATP metabolic process” (Figure 5A). Overrepresented molecular functions were “structural constituent of ribosome” and “rRNA binding” (Figure 5B). The most overrepresented cellular components were “cytosolic ribosome,” “cytosolic small and large ribosomal subunit,” “U5 snRNP,” “spliceosomal snRNP complex,” and “proteasome core complex” (Figure 5C). In the case of downregulated genes (485 genes), we did not observe GO terms that met the appropriate criteria.

We identified 165 common aging-related genes contributing to KEGG pathways. KEGG pathways were selected using the criteria -log(*p*-value) > 2.5. Figure 5D shows the gene number distribution for various KEGG pathways. Among the KEGG pathways, the most represented was “ribosome pathway,” followed by “degenerative diseases pathways such as Huntington’s disease, Alzheimer’s disease, and Parkinson’s disease,” “oxidative phosphorylation pathway,” “proteasome pathway,” “non-alcoholic fatty liver disease pathway,” and “spliceosome pathway.”

### Time series analysis and candidate aging genes

To investigate the global temporal transcriptional patterns, time series analysis was performed. We defined the DEGs across a time series as genes that are differentially expressed between two time points. Then, we performed time series expression profile clustering to identify common temporal expression patterns. DEGs were clustered into nine groups, i.e., up-up (29 genes), up-unchanged (1,034 genes), up-down (327 genes), unchanged-up (519 genes), unchanged-unchanged (21,977 genes), unchanged-down (167 genes), down-up (1,080 genes), down-unchanged (944 genes), and down-down (not detected) (Supplementary Figure 3). Based on the time series clustering, up-up cluster genes were selected as the candidate aging genes (Table 1). In particular, the changes in the expression of nine genes (*RPL37A, RPS21, RPL37, RPS26, RPS27A, RPL13A, GLRX3, RPL32,* and *TMA7*) showed a more cumulatively increased pattern than those in the other candidate aging genes.

**Table 1:** Candidate aging genes.

**Gene Name**	**Accession no.**	**Description**	**Chr**	**Srart**	**End**	**Strand**	**1^st^ DEGs**		**2^nd^ DEGs**	
							***p*-value**	**Log_2_FC**	***p*-value**	**Log_2_FC**
RPL37A	ENSCSAG00000000609	ribosomal protein L37a	1	82546802	82547077	-	0.0043	0.60	5.00E-05	1.15
RPS21	ENSCSAG00000016013	ribosomal protein S21	2	1841865	1843087	-	0.0076	0.81	5.00E-05	1.37
RPL37	ENSCSAG00000016909	60S ribosomal protein L37	4	39586974	39590110	-	5.00E-05	1.20	5.00E-05	1.80
RPL6	ENSCSAG00000018863	60S ribosomal protein L6	5	39187341	39188207	+	0.0001	1.24	0.00585	0.62
RPS26	ENSCSAG00000019637	ribosomal protein S26	6	40596460	40596804	-	0.0107	0.75	0.0002	0.83
BAX	ENSCSAG00000002776	BCL2-associated X protein	6	42186238	42194115	+	0.01115	0.58	0.0398	0.39
RPS27A	ENSCSAG00000000427	ribosomal protein S27a	7	2002541	2003068	+	0.0012	0.86	5.00E-05	1.07
H2AFZ	ENSCSAG00000000395	H2A histone family member Z	7	65001927	65002313	-	0.0064	0.66	0.0032	0.57
NDUFS5	ENSCSAG00000000098	NADH dehydrogenase (ubiquinone) Fe-S protein 5	7	117309172	117309649	+	5.00E-05	1.79	0.0014	0.79
CHCHD2	ENSCSAG00000018548	coiled-coil-helix-coiled-coil-helix domain containing 2, mitochondrial	8	53292182	53292871	+	0.03975	0.47	0.02065	0.44
RPL36	ENSCSAG00000018603	ribosomal protein L36	8	97021222	97021592	+	5.00E-05	0.90	0.0032	0.53
RPL13A	ENSCSAG00000019054	ribosomal protein L13a	9	13383840	13384448	-	0.046	0.48	0.00585	0.57
CHCHD1	ENSCSAG00000008922	coiled-coil-helix-coiled-coil-helix domain containing 1	9	57589462	57590554	-	0.0117	1.79	0.02235	0.78
GLRX3	ENSCSAG00000004419	glutaredoxin 3	9	122737379	122781391	+	0.03215	0.47	0.00305	0.55
MRPL51	ENSCSAG00000010565	mitochondrial ribosomal protein L51	11	6532431	6533536	-	0.0002	0.94	5.00E-05	0.82
RPL32	ENSCSAG00000018921	60S ribosomal protein L32	11	79856860	79857264	-	0.01655	0.55	5.00E-05	0.89
C12orf75	ENSCSAG00000002966	chromosome 12 open reading frame 75	11	100538647	100575739	+	0.0104	1.75	0.01225	1.04
MDH1	ENSCSAG00000014691	malate dehydrogenase 1, NAD	14	43406499	43425095	-	0.00365	0.58	0.01465	0.44
RPS4	ENSCSAG00000019456	40S ribosomal protein S4	14	69231717	69232508	-	5.00E-05	2.16	0.0125	0.62
SUPT4H1	ENSCSAG00000005761	suppressor of Ty 4 homolog 1 (S. cerevisiae)	16	35047934	35054239	+	0.0409	0.44	0.03495	0.40
COX7A2	ENSCSAG00000014488	cytochrome c oxidase subunit VIIa polypeptide 2 (liver)	17	45771	51669	+	0.00015	1.17	0.00025	0.83
TOMM6	ENSCSAG00000011289	Mitochondrial importreceptor subunit TOM6 homolog	17	30373701	30375892	-	0.0104	0.60	0.035	0.38
ATP5O	ENSCSAG00000019522	ATP synthase, H+ transporting, mitochondrial F1 complex, O subunit	21	116427583	116428215	-	0.00015	0.77	0.00285	0.54
TMA7	ENSCSAG00000018802	translation machinery associated 7 homolog (S. cerevisiae)	22	30956078	30956269	-	0.00775	1.06	5.00E-05	1.72
PFDN1	ENSCSAG00000012913	prefoldin subunit 1	23	42892401	42953491	-	0.02735	0.46	0.0103	0.45
MRPS14	ENSCSAG00000011897	mitochondrial ribosomal protein S14	25	54231643	54240472	+	0.00015	1.26	0.0118	0.66
RPL39	ENSCSAG00000019753	60S ribosomal protein L39	26	48140075	48140227	-	5.00E-05	4.37	0.0015	1.26
SNURF	ENSCSAG00000006401	SNRPN upstream reading frame protein	26	57098072	57103910	-	0.00915	1.31	0.0268	0.86
MRPL46	ENSCSAG00000017201	mitochondrial ribosomal protein L46	29	7025442	7033219	-	0.0371	0.51	0.03695	0.42

## DISCUSSION

To our knowledge, the longitudinal whole transcriptome study reported here is the first to examine aging in aged NHPs. In this study, we used aged AGMs to investigate the transcriptome over a period of two years to identify aging-related genes and potential aging-related pathways. Intriguingly, all our results indicated similar major biological processes, such as translation-associated ribosomal biogenesis, during normal aging. Specifically, 29 candidate aging genes with increased expression over time were identified. Our results also suggest that candidate aging genes may serve as new potential targets for aging or aging biomarkers.

Old World monkeys (e.g. Macaque species and AGMs) serve as robust aging models because they display a realistic aging course with time compression [22, 23]. AGMs have a relatively long lifespan (average, 20 years; maximum lifespan, ~31 years in captivity) [19]. In the case of rhesus monkeys (a Macaque species), the average lifespan in captivity is approximately 26 years [22, 24]. OWMs age in a manner similar to that observed in humans, but at a rate that is approximately three times faster than human aging [22]. Rhesus monkeys are generally considered old after approximately 20 years of age. Indeed, rhesus monkeys show significant signs of physical decline, such as reduced mobility and skin atrophy, by their late 20s [25]. At these later ages, they also develop many disorders common in older humans, including cancer, cataracts, osteopenia, and cardiovascular disease. Aging-related changes in OWMs more closely approximate similar changes observed in humans than those observed in shorter-lived aging models [18]. Though aging research using OWMs involves many difficulties, including high cost, specialized facilities, and NHP scientific specialists, OWMs provide a powerful aging model. Because of this, studies on aging using monkeys often have small sample sizes and are generally cross-sectional rather than longitudinal. Despite such difficulties, research on aging using OWMs remains the attractive translational approach to understand human aging, the mechanisms underlying aging, and aging-related disease [18]. In this study, we investigated the changes at the transcriptome level over a period of two years. For AGMs, two years are equivalent to nearly six to seven human years, so our AGM models can be considered equivalent to 50-70-year-old humans. During our study, we did not observe any other aging-related diseases except physical decline in our AGMs models. Thus, to our knowledge, we provide the first demonstration of transcriptome changes during normal aging using aged primate models.

While chronological age is the amount of time after birth, biological age is measured by physical or functional assessments. Biological age is influenced by various factors, including nutrition, stress, lifestyle, disease, and genetic background [3]. Although chronological age is the same, biological age could be different. Because of the different rates of aging, it is difficult to identify accurate indicators of aging using chronological age alone [26]. In longitudinal studies, serial samples allow comparisons in the same individual at different time points, rather than among other individuals at different ages. Thus, longitudinal studies that observe the same individual throughout their lifespan provide better understanding of aging. Our longitudinal study using aged AGM model allowed us to control for numerous environmental factors, so we could accurately analyze the changes in gene expression over time.

Previous studies suggest a direct connection between dysregulated ribosome biogenesis and aging. For instance, attenuated protein synthesis via caloric restriction or genetic manipulation of ribosome biogenesis-related genes is known to increase the lifespan of multiple organisms, including *C. elegans*, mice, and humans [27–29]. Therefore, promoting ribosome biogenesis could accelerate aging. Indeed, a recently published study revealed increased ribosomal biogenesis and activity as hallmarks of premature aging in human fibroblasts [30]. In our study, gene expression was clustered relatively well at each time point (Figure 1). This result suggests the existence of gene groups involved in the aging process over time. Indeed, we identified individual aging-related DEGs and common aging-related DEGs. The findings from the GO enrichment analyses indicated enrichment of biological processes involved in ribosome biogenesis and translation (Figures 3, 5). This is consistent with the finding that cytosolic ribosomal proteins are upregulated with age in various human tissues, including brain [31], kidney [32], and muscle [33]. Moreover, a 10-year longitudinal study demonstrated that a similar number of DEGs are expressed between the ages of 70 and 80 in human whole blood [8]. These DEGs showed significant enrichment for multiple aging-related pathways, including protein metabolism and oxidative phosphorylation. Many of these processes have been previously described in aging [30, 34, 35]. Individual GO terms were “RNA splicing” and “protein folding associated with protein quality control” (Figure 3A). These results suggest that the processes involved in common natural aging are associated with ribosome biogenesis and translation, and those involved in individual aging are associated with RNA splicing, protein folding, and protein quality control. Moreover, 29 candidate aging genes were associated with ribosomes, cytoplasmic translation, and mitochondrial translational termination. The expression of these genes in whole blood was consistently associated with these processes, with increased gene expression over the period of two years (Table 1). Increased expression of ribosome and translation-associated genes, including the 29 aging candidate genes identified in this study, appears to be a potential biomarker of aging that requires further functional analysis.

Several hypotheses have been suggested to explain the process of aging. Some gene-centric theories have been suggested; these primarily focus on harmful changes in the genome that accumulate during the life cycle [36], including DNA mutation and shortened telomeres. Other epigenetic theories have been suggested; these focus on how changes in the DNA and DNA-binding proteins affect gene expression [37]. These epigenetic changes include DNA methylation, histone modification, and loss of chromosomal organization. Other theories of aging suggest declining quality of control systems during protein synthesis and degradation and chaperone systems [38]. The decline in protein quality control is involved in abnormal protein accumulation, because protein production and disposal become increasingly compromised with age. However, we already know that due to the complexity of the aging process, an integrated approach is needed to better understand the mechanism of aging. In this respect, genomics, transcriptomics, epigenomics, proteomics, and metabolomics could provide crucial evidence for explaining the complex and interconnected changes that occur during aging, though current knowledge of these molecular interactions is still limited.

**Figure 6:**
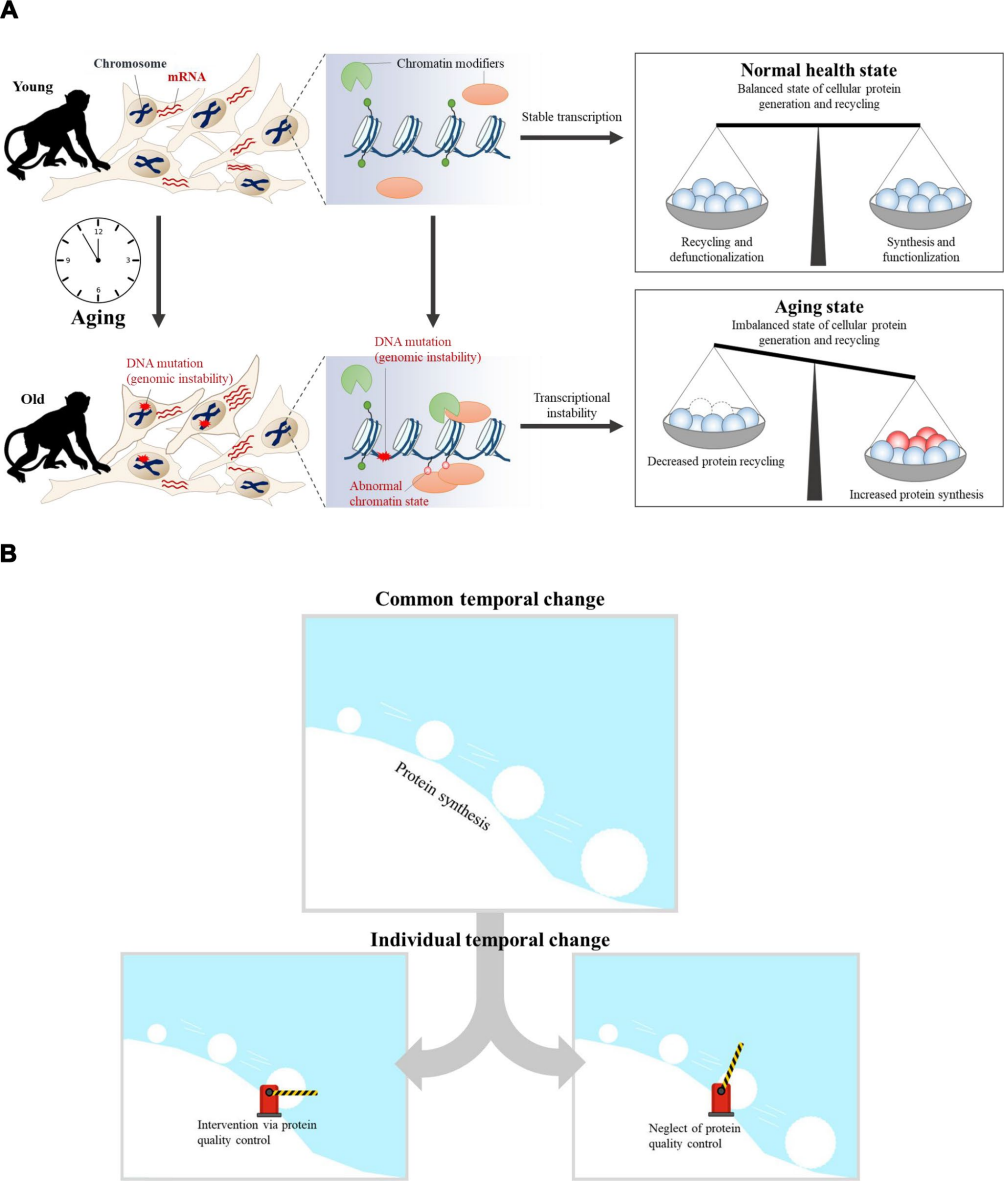
**The aging snowball effect model.** (**A**) In the case of young cells, transcriptional programs are tightly controlled by epigenetic regulators. As a result, balanced cellular protein synthesis and recycling are maintained in the ‘normal healthy state.’ However, transcriptional instability increases concomitantly with age. The accumulation of DNA mutations can trigger the recruitment of chromatin modifiers, which results in abnormal chromatin structure and transcriptional instability. Thus, the ‘aged state’ becomes an imbalanced state wherein cellular protein synthesis and recycling are dysregulated. (**B**) Aging is the result of accumulated dysregulation and damage that results in a “snowball” effect. Accumulated dysregulation and damage is promoted by upregulated protein synthesis during aging. Upregulated protein synthesis has an increasingly greater impact on cellular aging, similar to a snowball rolling down a hill. Individual aging processes could be affected by targeting protein quality control systems, provided that this is the common aging process.

Previous research showed that errors in DNA repair and inaccurate replication lead to the accumulation of DNA mutations and epimutations with age [39, 40]. Genomic instability via accumulated DNA damage in aging may also promote specific epigenetic alterations, such as global loss of chromatin compaction through the recruitment of chromatin modifiers, including Polycomb, *SIRT1*, *SIRT6*, and methyltransferases [40]. The accumulation of epimutations via chromatin modifications in aging may also induce changes in gene expression, thus inducing transcriptional instability [4]. Although we have focused on temporal transcriptome changes with aging in this study, we suggest a hypothesis called the “snowball effect via ribosomal biogenesis,” when considered with previous aging studies [41] (Figure 6). In this model, alterations in ribosomal biogenesis play a role in gradually increasing the effects on aging. The accumulation of DNA mutations induces epimutations during aging, thereby inducing transcriptional instability. Because the rate of protein translation is proportional to the rate of ribosome biogenesis, upregulated protein synthesis is related to disrupted global proteostasis [42]. While a normal healthy state maintains balanced cellular protein synthesis and recycling, aging induces an imbalanced state of protein synthesis and recycling (Figure 6A). In the common aging process, upregulated protein synthesis exhibits an increasingly greater impact on cellular aging, similar to a snowball rolling down a hill. Some individual aging processes would be affected via interventions of protein quality control systems (Figure 6B). Intervention of protein quality control systems would have induced individual difference in aging.

In summary, we present the first longitudinal characterization of transcriptional changes in aging NHPs. Our findings indicate that translation-related genes, such as those involved in ribosome biogenesis, are upregulated in normal aging. We identified 29 candidate aging genes that could serve as an attractive target for the treatment of aging, or could function as biomarkers for aging. However, this is just the beginning of our understanding of the aging process. To substantially explain our aging hypothesis, our next step involves a comprehensive approach to collect integrated -omics evidence using genomics, transcriptomics, and epigenomics.

## MATERIALS AND METHODS

### Ethical approval

All animal procedures were conducted based on the Guidelines of the Institutional Animal Care and Use Committee (KRIBB-AEC-14007, KRIBB-AEC-15031, and KRIBB-AEC-16067) at the Korea Research Institute of Bioscience and Biotechnology (KRIBB).

### Animals and sampling

Nine naturally-aged AGMs were used in this study. Their characteristics are summarized in Supplementary Table 1. All animals were provided by the National Primate Research Center (NPRC) of Korea. In our experiments, specific pathogen-free (SPF) animals were used. All animals were subjected to a complete physical assessment, including viral, bacterial, and parasite examinations. On physical examination, SPF animals were examined for coat condition, appearance, weight, sex, and date of birth. Enzyme immunoassays were performed to detect viruses, such as simian herpes B virus (BV), simian T-cell lymphotropic/leukemia virus (STLV)-1 and -2, simian immunodeficiency virus (SIV), simian retrovirus (SRV)-1, -2, and -5, and simian virus 40 (SV40). In addition, tests were performed to detect *Mycobacterium tuberculosis* (TB), *Shigella* spp., *Salmonella* spp., and pathogenic *E. coli*. In our SPF animals, all these tests were negative. The monkeys were housed indoors in individual cages and fed commercial monkey chow2 (Harlan) supplemented daily with various fruits, and supplied water *ad libitum*. Environmental conditions were controlled to provide a constant temperature of 24 ± 2° C, 50 ± 5% relative humidity, 100% fresh air at ≥12 room changes/h, and a 12 h light:dark cycle. Each monkey was given access to environmental enrichment such as approved toys, perches, or music to promote psychological well-being. Animal health was monitored by the attending veterinarian consistent with the recommendations of the Weatherall Report. Nine naturally aged AGMs were exposed in the same environment during the study. These monkeys were in individual cages of the same indoor room and were provided the same food conditions, such as commercial monkey feed and fruits.

Peripheral blood samples from nine aged AGMs of varying ages from 15-23 years old were collected three times over a period of two years (Supplementary Table 1). The collected whole blood samples were analyzed the cell type composition by hematology analyzer (Hemavet950, Drew Scientific, USA) (Supplementary Figure 4). We performed paired *t*-test to considerate about potential confounding effects from collected blood samples at each time point. As a result of paired *t*-test, there was no statistical difference in blood cell counts between each group except monocyte at TP1 vs TP2 (Supplementary Table 4). Furthermore, blood samples were collected by venipuncture and stored in PAXgene tubes (PreAnalytiX, Hombrechtikon, Switzerland) for RNA extraction and sequencing. Peripheral blood sampling processes and methods were always same at each time-point.

### RNA extraction and high-throughput paired-end RNA sequencing

Total RNA was extracted using PAXgene Blood RNA Kits (Qiagen, Hilden, Germany). RNase-free DNase (Qiagen, GmbH, Hilden, Germany) was used to eliminate DNA contamination from the total RNA preparations. The quality of the prepared total RNA was evaluated using an Agilent 2100 Bioanalyzer (Agilent, USA). Each total RNA had an RNA integrity number (RIN) >7.5. cDNA libraries for RNA-seq were prepared from RNA samples using oligo d(T) primer. Constructed libraries were sequenced using an Illumina HiSeq 2000 sequencer (Illumina, USA). We performed paired *t*-test to considerate about potential confounding effects from RNA extraction and RNA sequencing of samples at each time point. As a result of paired *t*-test, there was no statistical difference in total RNA quality between each group (Supplementary Table 5). Although, RNA extraction, library preparation, and sequencing were performed at each time-point, were always performed by the same technician to minimize confounding effects.

### RNA-seq data processing

Images generated by the HiSeq 2000 were converted into nucleotide sequences by a base-calling pipeline and stored in FASTQ format. Low-quality reads were removed prior to mapping and assembly. Three criteria were used to filter out raw dirty reads, i.e., 1) reads with more than 10 % ‘N’ bases; 2) low-quality reads which had more than 40 % QA ≤ 20 bases; and 3) reads with an average quality score < 20. All subsequent analyses used clean reads.

Clean reads were mapped to the reference *Chlorocebus sabaeus* transcriptome sequences from the Ensembl database (ChlSab1.1), using Bowtie2 and Tophat 2.0.1. Mismatches ≤ 3 base pairs were allowed in each read alignment. FPKM values for each gene were calculated, and DEGs were identified using this value as described in the Results section. For genes with more than one alternative transcript, the longest transcript was used to calculate the FPKM value.

### Gene expression analysis

Hierarchical clustering analysis was performed using Cluster 3.0. All FPKM values were increased by 1 and log_2_ transformed. The data were adjusted by centering genes at the median. Clustering was performed using differential distance metrics and average linkage as the method. TreeView Software was used to visualize the clustering results. Differential gene expression analysis was performed using Cuffdiff. The differential expression *p*-values were adjusted using the Benjamini and Hochberg procedure, resulting in the false discovery rate (FDR), which was set to an FDR < 0.05 cutoff. Venn diagrams were constructed using online Venny software (http://bioinfogp.cnb.csic.es/tools/venny/) to identify aging-associated genes. Then, aging-associated genes were defined as the intersecting DEGs between each time point. For time series expression analysis, *p*-values were used as the main filter to identify genes with differential expression across the time points. Therefore, genes with log_2_FC > 0 and *p*-value < 0.05 were classified as being upregulated, while genes with log_2_FC < 0 and *p*-value < 0.05 were classified as being downregulated.

### Functional annotation

GO slim terms were analyzed using the PANTHER Classification System (http://www.pantherdb.org/) version 14.0. Functional annotation and KEGG pathway analysis were performed using DAVID (Database for Annotation, Visualization, and Integrated Discovery, version 6.8; http://david.abcc.ncifcrf.gov).

## Supplementary Material

Supplementary Figures

Supplementary Tables 1 and 2

Supplementary Table 3

Supplementary Tables 4 and 5
