# Supplementary material for: Longitudinal profiling of the blood transcriptome in an African green monkey aging model
Source: Aging (Albany NY). 2020 Dec 3;13(1):846–64. doi: 10.18632/aging.202190 (PMC7834999; doi:10.18632/aging.202190)
Supplement: Supplementary Figures [file aging-13-202190-s001.pdf]

SUPPLEMENTARY FIGURES

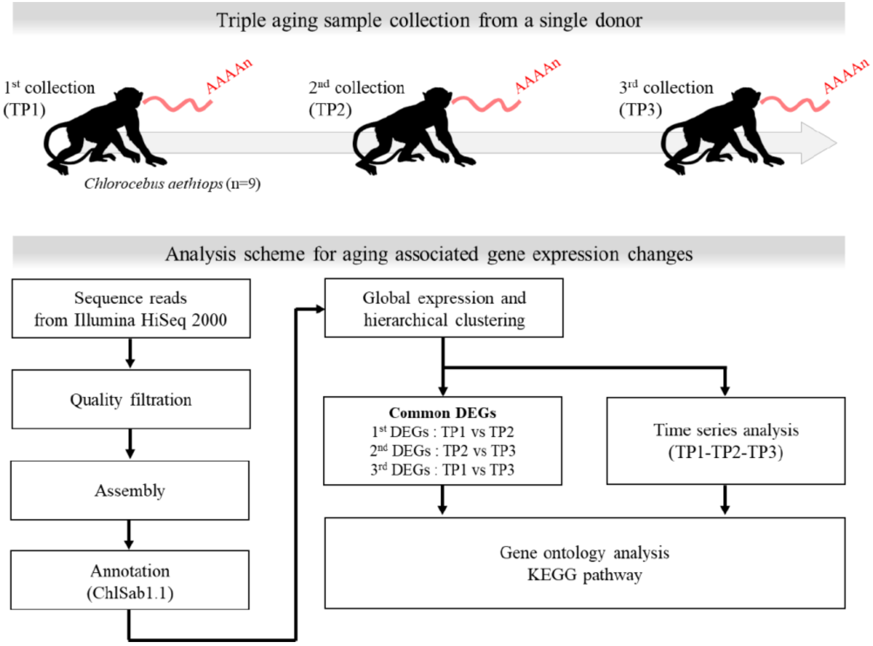

Supplementary Figure 1. Overview of the experimental design and data analysis steps used for the study.

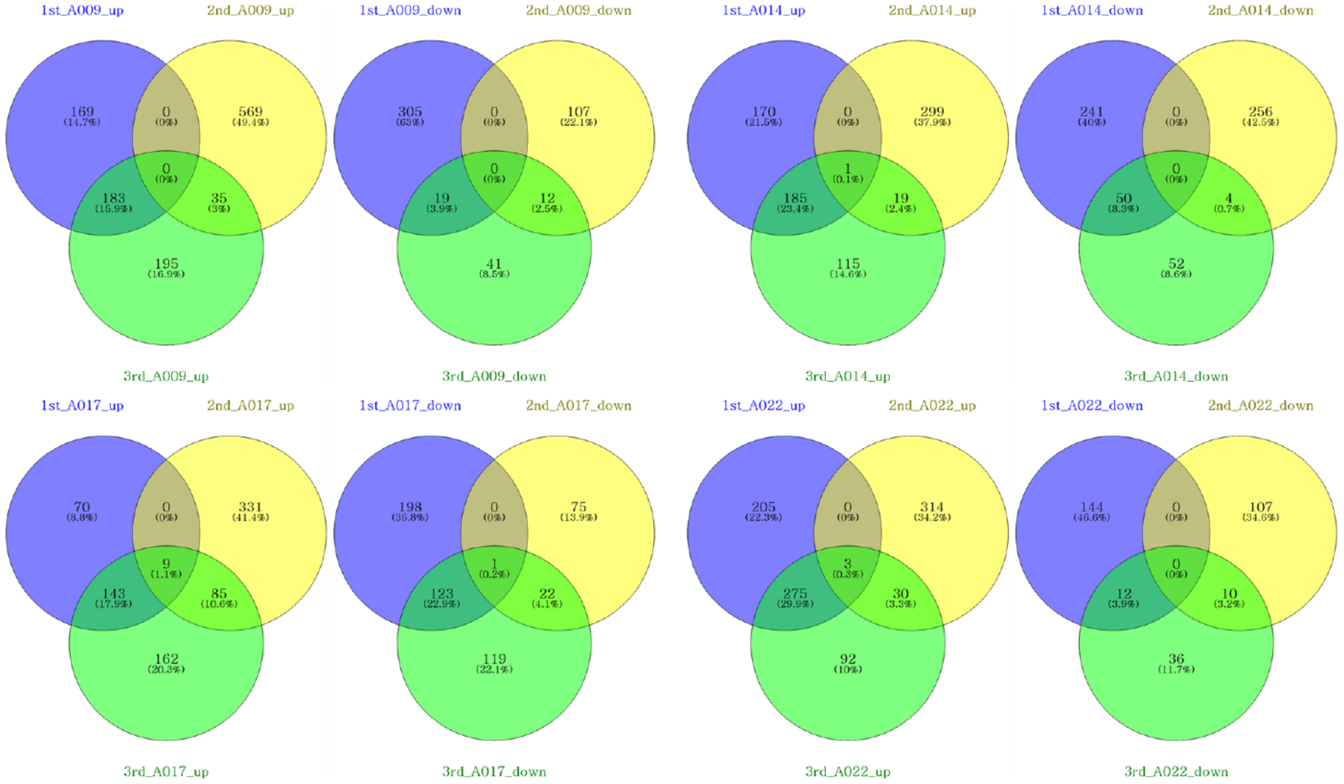

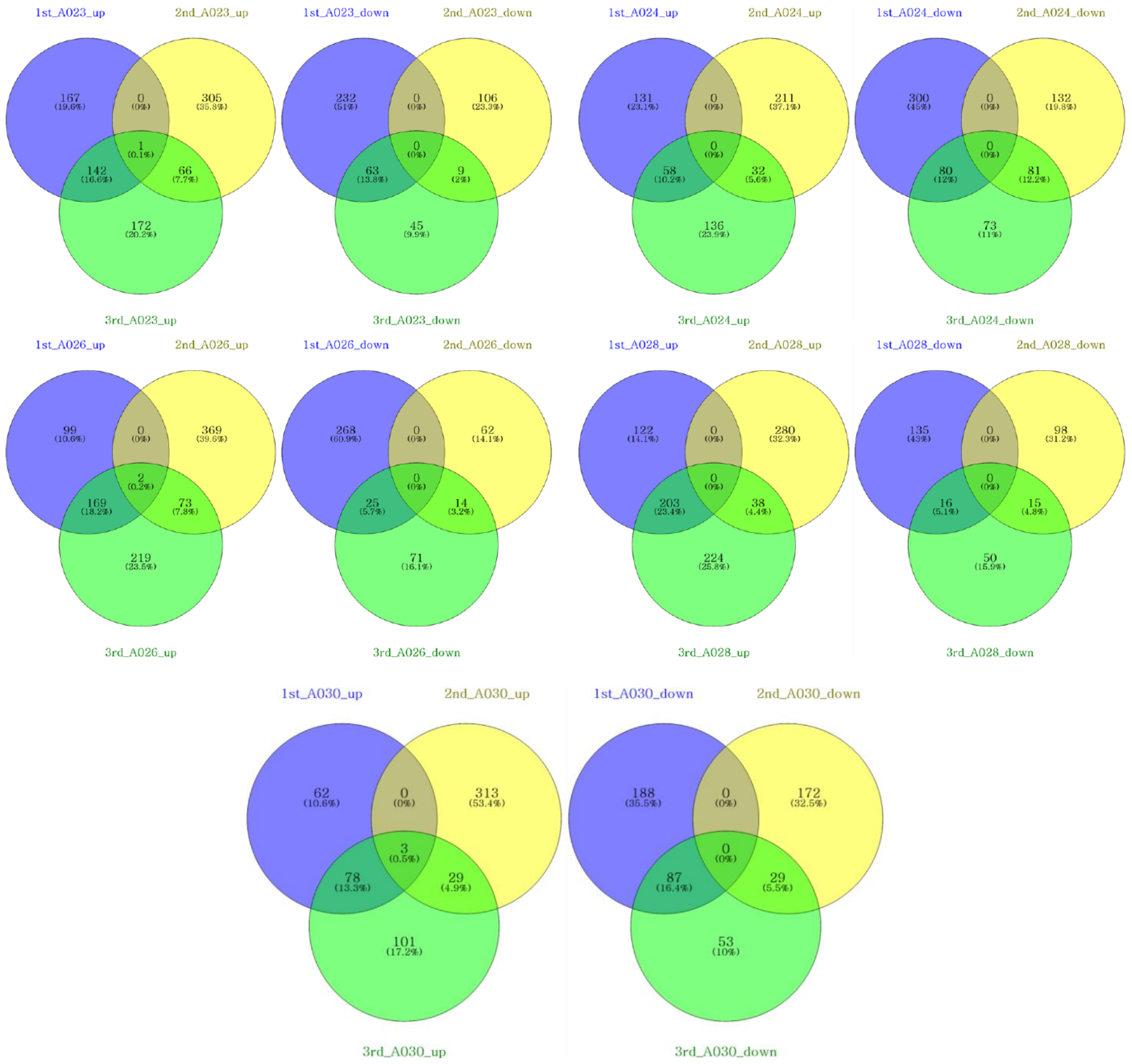

Supplementary Figure 2. Venn diagrams showing the overlap of differentially expressed genes (DEGs) from pairwise comparisons of each time point in each individual. Venn diagrams were constructed using Venny online software.

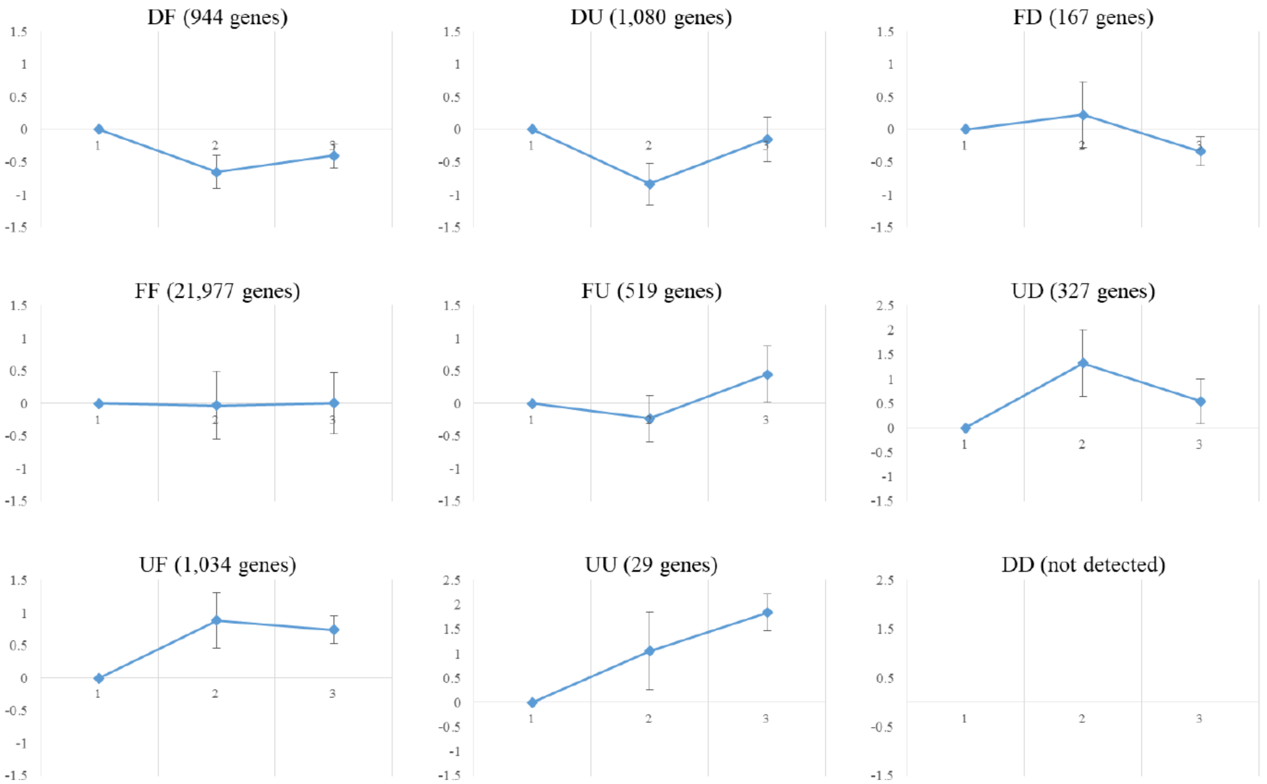

Supplementary Figure 3. Time series DEG analysis. DEGs were clustered into nine groups according to differential expression patterns across a time series, i.e., up-up (29 genes), up-unchanged (1,034 genes), up-down (327 genes), unchanged-up (519 genes), unchanged-unchanged (21,977 genes), unchanged-down (167 genes), down-up (1,080 genes), down-unchanged (944 genes), and down-down (not detected). Time points are plotted on the X-axis, and average fold change is plotted on the Y-axis.

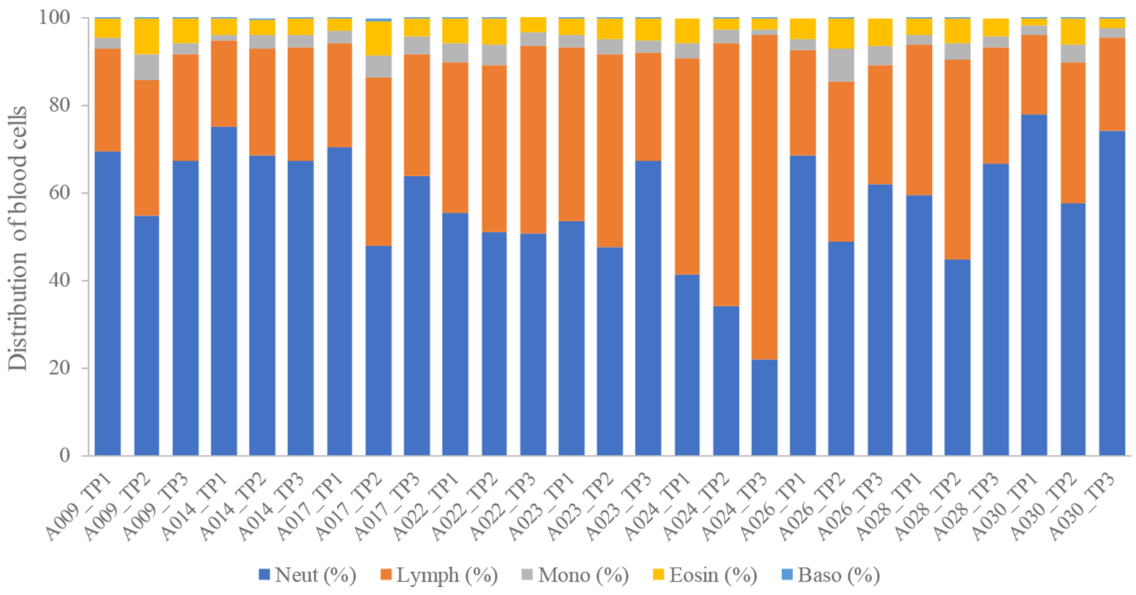

Supplementary Figure 4. Distribution of blood cells. Cell type frequency estimates (in %). Triple pairs of columns were three time-points samples of the same individual.
