# Supplementary material for: Longitudinal profiling of the blood transcriptome in an African green monkey aging model
Source: Aging (Albany NY). 2020 Dec 3;13(1):846–64. doi: 10.18632/aging.202190 (PMC7834999; doi:10.18632/aging.202190)
Supplement: Supplementary Tables 1 and 2 [file aging-13-202190-s002.pdf]

SUPPLEMENTARY TABLES

Supplementary Table 1. Sample information used in this study.

Species	Identical No.	Sex (F/M)	Birth	1st year (TP1) Age	2nd year (TP2) Age	3rd year (TP3) Age
Chlorocebus aethiops	A009	M	February 5, 1991	23	24	25
	A014	F	April 20, 1992	22	23	24
	A017	F	August 8, 1992	22	23	24
	A022	F	November 12, 1993	21	22	23
	A023	F	December 18, 1993	21	22	23
	A024	F	March 27, 1994	20	21	22
	A026	F	June 16, 1994	20	21	22
	A028	F	August 27, 1995	19	20	21
	A030	M	October 1, 1999	15	16	17

Supplementary Table 2. Summary of the whole-transcriptome sequencing data used in this study.

Sample	Total read number	Clean reads	Clean bases	% >=Q20 bases	GC(%)	Uniquely mapped reads	Mapping rate (%)
A009_TP1	45,301,314	43,323,392 (95.6%)	4,332,339,200	98.76%	55.74%	36,488,069	84.2%
A014_TP1	47,470,650	45,024,446 (94.8%)	4,502,444,600	98.71%	55.90%	37,676,100	83.7%
A017_TP1	46,834,102	44,680,648 (95.4%)	4,468,064,800	98.77%	54.81%	38,165,871	85.4%
A022_TP1	46,924,778	44,894,386 (95.7%)	4,489,438,600	98.80%	54.55%	39,893,408	88.9%
A023_TP1	59,568,668	56,200,418 (94.3%)	5,620,041,800	98.47%	55.45%	48,230,648	85.8%
A024_TP1	49,561,172	47,576,264 (96.0%)	4,757,626,400	98.81%	56.02%	39,524,017	83.1%
A026_TP1	51,322,386	48,434,668 (94.4%)	4,843,466,800	98.50%	53.54%	41,257,942	85.2%
A028_TP1	44,904,020	42,838,080 (95.4%)	4,283,808,000	98.72%	55.15%	36,417,864	85.0%
A030_TP1	48,547,742	46,015,192 (94.8%)	4,601,519,200	98.59%	58.99%	37,872,906	82.3%
A009_TP2	57,665,986	56,416,626 (97.8%)	5,698,079,226	97.49%	56.26%	52,126,315	92.4%
A014_TP2	59,140,718	57,749,646 (97.6%)	5,832,714,246	97.69%	56.76%	52,795,462	91.4%
A017_TP2	54,164,580	52,825,084 (97.5%)	5,335,333,484	97.32%	51.93%	45,950,900	87.0%
A022_TP2	54,043,820	52,595,934 (97.3%)	5,312,189,334	97.12%	54.43%	46,766,815	88.9%
A023_TP2	55,098,080	53,515,516 (97.1%)	5,405,067,116	97.15%	55.58%	47,224,418	88.2%
A024_TP2	62,305,174	60,598,090 (97.3%)	6,120,407,090	97.23%	54.68%	54,512,504	90.0%
A026_TP2	60,312,888	58,368,442 (96.8%)	5,895,212,642	96.91%	51.75%	50,263,252	86.1%
A028_TP2	58,472,078	56,823,852 (97.2%)	5,739,209,052	97.24%	55.50%	50,034,000	88.1%
A030_TP2	54,537,530	52,875,002 (97.0%)	5,340,375,202	97.16%	55.24%	47,169,159	89.2%
A009_TP3	58,391,966	57,430,426 (98.4%)	5,800,473,026	97.94%	56.96%	50,712,008	88.3%
A014_TP3	43,120,278	42,268,894 (98.0%)	4,269,158,294	97.82%	55.95%	37,376,907	88.4%
A017_TP3	50,063,882	49,285,080 (98.4%)	4,977,793,080	97.99%	55.34%	43,765,097	88.8%
A022_TP3	58,666,010	57,690,134 (98.3%)	5,826,703,534	97.89%	56.70%	50,868,104	88.2%
A023_TP3	51,392,162	50,689,146 (98.6%)	5,119,603,746	97.28%	55.87%	44,505,333	87.8%
A024_TP3	51,849,460	51,032,314 (98.4%)	5,154,263,714	97.97%	57.34%	45,655,035	89.5%
A026_TP3	52,834,220	52,040,756 (98.5%)	5,256,116,356	97.24%	54.81%	45,127,840	86.7%
A028_TP3	50,839,704	50,039,096 (98.4%)	5,053,948,696	97.98%	56.48%	44,695,945	89.3%
A030_TP3	59,264,442	58,377,208 (98.5%)	5,896,098,008	98.00%	57.34%	52,561,099	90.0%
