# Supplementary material for: Longitudinal profiling of the blood transcriptome in an African green monkey aging model
Source: Aging (Albany NY). 2020 Dec 3;13(1):846–64. doi: 10.18632/aging.202190 (PMC7834999; doi:10.18632/aging.202190)
Supplement: Supplementary Table 3 [file aging-13-202190-s003.pdf]

Supplementary Table 3

Table S3. Pearson's correlation coefficient in each sample

	A009 TP1	A014 TP1	A017 TP1	A022 TP1	A023 TP1	A024 TP1	A026 TP1	A028 TP1	A030 TP1	A009 TP2	A014 TP2	A017 TP2	A022 TP2	A023 TP2	A024 TP2	A026 TP2	A028 TP2	A030 TP2	A009 TP3	A014 TP3	A017 TP3	A022 TP3	A023 TP3	A024 TP3	A026 TP3	A028 TP3	A030 TP3
**A009_TP1**	-																										
**A014_TP1**	0.924	-																									
**A017_TP1**	0.813	0.82	-																								
**A022_TP1**	0.942	0.932	0.764	-																							
**A023_TP1**	0.949	0.922	0.775	0.938	-																						
**A024_TP1**	0.775	0.755	0.533	0.741	0.8	-																					
**A026_TP1**	0.924	0.872	0.883	0.918	0.896	0.655	-																				
**A028_TP1**	0.941	0.938	0.775	0.94	0.951	0.804	0.881	-																			
**A030_TP1**	0.864	0.85	0.599	0.816	0.875	0.843	0.726	0.871	-																		
**A009_TP2**	0.789	0.715	0.635	0.741	0.774	0.685	0.744	0.744	0.734																		
**A014_TP2**	0.823	0.828	0.745	0.796	0.834	0.717	0.788	0.8	0.786	0.9	-																
**A017_TP2**	0.816	0.804	0.878	0.766	0.801	0.581	0.88	0.784	0.621	0.786	0.859	-															
**A022_TP2**	0.859	0.798	0.7	0.833	0.85	0.705	0.853	0.815	0.738	0.916	0.916	0.852	-														
**A023_TP2**	0.835	0.771	0.764	0.799	0.833	0.652	0.84	0.792	0.717	0.884	0.89	0.878	0.936	-													
**A024_TP2**	0.837	0.807	0.607	0.75	0.83	0.818	0.788	0.85	0.792	0.901	0.894	0.726	0.872	0.883	-												
**A026_TP2**	0.818	0.761	0.755	0.743	0.81	0.623	0.876	0.789	0.665	0.844	0.853	0.916	0.891	0.893	0.785	-											
**A028_TP2**	0.863	0.827	0.713	0.839	0.864	0.773	0.819	0.878	0.805	0.922	0.911	0.847	0.929	0.925	0.951	0.889	-										
**A030_TP2**	0.822	0.785	0.709	0.787	0.844	0.736	0.808	0.808	0.802	0.899	0.912	0.845	0.928	0.912	0.891	0.871	0.919	-									
**A009_TP3**	0.903	0.815	0.738	0.836	0.864	0.739	0.855	0.826	0.807	0.879	0.857	0.8	0.895	0.882	0.873	0.846	0.88	0.866									
**A014_TP3**	0.885	0.896	0.694	0.851	0.894	0.74	0.827	0.855	0.824	0.827	0.923	0.747	0.875	0.845	0.829	0.777	0.873	0.875	0.897	-							
**A017_TP3**	0.842	0.807	0.759	0.745	0.842	0.678	0.865	0.808	0.734	0.842	0.871	0.857	0.848	0.891	0.768	0.847	0.875	0.89	0.896	0.865	-						
**A022_TP3**	0.89	0.835	0.72	0.868	0.877	0.786	0.836	0.856	0.822	0.878	0.884	0.802	0.926	0.885	0.91	0.849	0.917	0.894	0.946	0.925	0.909	-					
**A023_TP3**	0.867	0.841	0.812	0.82	0.886	0.697	0.849	0.842	0.75	0.821	0.873	0.856	0.855	0.925	0.796	0.841	0.879	0.874	0.905	0.866	0.883	0.907	-				
**A024_TP3**	0.782	0.743	0.58	0.752	0.796	0.906	0.71	0.791	0.837	0.795	0.786	0.634	0.786	0.762	0.894	0.696	0.843	0.795	0.839	0.816	0.759	0.875	0.761	-			
**A026_TP3**	0.844	0.789	0.749	0.816	0.823	0.671	0.885	0.785	0.736	0.81	0.824	0.831	0.822	0.87	0.729	0.88	0.841	0.848	0.912	0.827	0.885	0.898	0.896	0.755	-		
**A028_TP3**	0.875	0.836	0.692	0.841	0.875	0.772	0.83	0.876	0.82	0.836	0.85	0.757	0.871	0.869	0.867	0.804	0.922	0.867	0.924	0.929	0.879	0.943	0.903	0.859	0.869	-	
**A030_TP3**	0.791	0.726	0.634	0.736	0.803	0.771	0.731	0.754	0.823	0.847	0.827	0.71	0.835	0.794	0.842	0.751	0.848	0.878	0.903	0.878	0.873	0.912	0.841	0.881	0.839	0.893	-

**Corrrelation Ranges**	1.0~0.92	0.92~0.80	0.80~0.70	0.70~0.00
