# Supplementary material for: Longitudinal profiling of the blood transcriptome in an African green monkey aging model
Source: Aging (Albany NY). 2020 Dec 3;13(1):846–64. doi: 10.18632/aging.202190 (PMC7834999; doi:10.18632/aging.202190)
Supplement: Supplementary Tables 4 and 5 [file aging-13-202190-s004.pdf]

SUPPLEMENTARY TABLES

Supplementary Table 4. Summary of paired *t*-test results for blood cell types.

Cell types	*p*-value (Paired *t*-test)		
	TP1 vs. TP2	TP1 vs. TP3	TP2 vs. TP3
Neutrophil	0.594	0.901	0.681
Eosinophil	0.058	0.801	0.058
Basophil	0.240	0.624	0.167
Lymphcyte	0.065	0.418	0.420
Monocyte	0.022	0.443	0.096

Supplementary Table 5. Summary of paired *t*-test results for total RNA quality.

Total RNA quality	*p*-value (Paired *t*-test)		
	TP1 vs. TP2	TP1 vs. TP3	TP2 vs. TP3
RIN	0.806	0.403	0.486
Concentration	0.497	0.519	0.279
